# Emergency tracheal intubation peri-operative risk factors and prognostic impact after esophagectomy

**DOI:** 10.1186/s12871-022-01918-9

**Published:** 2022-12-01

**Authors:** Jun-Le Liu, Jian-Wen Jin, Li-Li Lin, Zhong-Meng Lai, Jie-Bo Wang, Jian-Sheng Su, Liang-Cheng Zhang

**Affiliations:** 1 Department of anesthesiology, Union Hospital, Fujian Medical University, XinQuan Road 29th, 350001 Fuzhou, Fujian China; 2 Department of Clinical Medicine, Fujian Health College, 366th GuanKou, 350101 Fuzhou, Fujian China

**Keywords:** Esophageal cancer, Esophagectomy, Emergency tracheal intubation, Overall survival

## Abstract

**Background:**

Emergent endotracheal intubation (ETI) is a serious complication after Oesophagectomy. It is still unclear that perioperative risk factors and prognosis of these patients with ETI.

**Methods:**

Between January 2015 and December 2018, 21 patients who received ETI after esophagectomy were enrolled (ETI group) at the department of thoracic surgery, Fujian Union hospital, China. Each study subject matched one patient who underwent the same surgery in the current era were included (control group). Patient characteristics and perioperative factors were collected.

**Results:**

Patients with ETI were older than those without ETI (*p* = 0.022). The patients with history of smoking in ETI group were significantly more than those in control group (*p* = 0.013). The stay-time of postanesthesia care unit (PACU) in ETI group was significantly longer than that in control group (*p* = 0.001). The incidence of anastomotic leak or electrolyte disorder in ETI group was also higher than that in control group (*p* = 0.014; *p* = 0.002). Logistic regression analysis indicated history of smoke (HR 6.43, 95%CI 1.39–29.76, *p* = 0.017) and longer stay time of PACU (HR 1.04, 95%CI 1.01–1.83, *p* = 0.020) both were independently associated with higher risks of ETI. The 3-year overall survival (OS) rates were 47.6% in patients with ETI and 85.7% in patients without ETI (HR 4.72, 95%CI 1.31-17.00, *p* = 0.018). COX regression analysis indicated ETI was an independent risk factor affecting the OS.

**Conclusion:**

The study indicated that history of smoking and longer stay-time in PACU both were independently associated with higher risks of ETI; and ETI was an independent risk factor affecting the OS of patients after esophagectomy.

**Trial registration:**

This trial was retrospectively registered with the registration number of ChiCTR2000038549.

## Introduction

Esophageal cancer is an aggressive malignancy associated with suboptimal overall survival (OS), with 456,000 patients diagnosed each year worldwide [1]. Esophagectomy is the standard approach for the curative management of locally advanced esophageal cancer but is associated with a considerable number of complications even after its recent modification to a minimally invasive version [2]. The most common post-operative complications include pneumonia, anastomotic leakage, and atrial fibrillation [3, 4]. If persistent severe dyspnea, hypoxemia, or hemodynamic instability worsens, emergency tracheal intubation (ETI) must be performed to stabilize the critical vital signs of the patient.

A 4-year retrospective study has found that the 30-day mortality of inpatients who have undergone ETI is as high as 66.8% [5]. Among them, the mortality of surgical patients after ETI was 55.0%, while that of medical patients was as high as 77.0%. The reason for the high mortality of medical patients included that there were more patients in the end-stage, while surgical patients with ETI were often related to surgical factors.In fact, the prognosis of surgical patients strongly depends on the extent of the disease as well as on the general condition of the patient. Therefore, it may be important to predict post-operative complications and implement precautions accordingly. Chun Chen has shown that pre-operative sarcopenia is an independent risk factor for pulmonary infection after minimally invasive esophagectomy [6]. And pre-operative nutritional screening of patients who will undergo esophagectomy is highly advised [7]. Lucas Goense has indicated that tumor characteristics, peri-operative treatment, comorbidities, nutritional status, and cardiopulmonary function of patients are all closely related to prognosis in esophageal cancer [2]. It is known that hemodynamic instability in frail patients can decrease the blood flow to the site of anastomosis, causing gastric conduit ischemia and anastomotic leakage.

Several recent studies have shown that complications post-esophagectomy impair patient survival. Recognition of the influences of peri-operative clinical parameters on the occurrence of the most serious post-esophagectomy complication, ETI, might contribute to the improvement of peri-operative decision-making and the development of preventative approaches. However, such influences have not been characterized yet.

This study aimed to analyze the risk factors for post-esophagectomy ETI and the risk factors affecting the OS in relevant cases.

## Methods

### Study population

This study comprised all the 21 patients who received ETI after esophagectomy for various reasons at the Hospital, between January 2015 and December 2018 as the study subjects. Likewise, each study subject matched one patient who underwent esophagectomy in the study as control subject.

The matching criteria included (all cases undergone esophagectomy were arranged according to the date of operation): (1) The same surgeons; (2) The same surgical methods; (3) Respectively select the previous case of the case who received ETI as the control, and if the selected case also received ETI, continued to push forward one case as the control. This study was conducted in accordance with the amended Declaration of Helsinki. Before data collection, the Research Ethics Committee of the hospital approved this study and waived the requirement for informed consent (ChiCTR2000038549; 23/09/2020 of first registration). The study was conducted by two independent investigators, and any disagreements were resolved by a third investigator.

### Anesthesia

All the patients received prophylactic antibiotics (cefotaxime 1.0 g) 30 min before incision. After establishing the systems for monitoring the vital signs, such as invasive arterial blood pressure (iBP), venous oxygen saturation (SPO_2_), heart rate, and body temperature, anesthesia was performed via intra-venously administered midazolam, propofol, etomidate, sufentanil, cisatracurium, or rocuronium. Mechanical ventilation was performed using a left-sided double-lumen tube to enable desufflation of the operative lung during the thoracic phase of the surgery. During the single-lung ventilation step, a pressure-controlled ventilation strategy with a maximum pressure of 28 centimeters of water and tidal volume of 5–6 mL/kg was used. During the double-lung ventilation step, tidal volumes were set at 7–8 mL/kg to maintain the end-tidal CO_2_ at 35–45 mmHg. Anesthesia was maintained using sevoflurane (0.4 MAC), remifentanil (0.15–0.30 µg/kg/min), and propofol infusion (target-controlled infusion of 0.6–2.0 µg/mL) with 60–100% oxygen. The infusion rates of propofol and remifentanil varied according to the clinical judgment at the time and to achieve a target bispectral index of 40–60.

After the operation, when the patients with a rhythmic spontaneous breath were considered cardiorespiratory-stable, extubation in the post-anesthesia care unit (PACU) was encouraged, and then the patients were transferred to the surgical ward. If the patients could not be extubated due to insufficient ventilation, hypoxemia, or hemodynamic instability, the double-lumen endotracheal tube was replaced with a single-lumen endotracheal tube before they were transferred to the intensive care unit or surgical ward for further treatments.

The post-operative pain management was maintained via intra-venous patient-controlled analgesia (3.0 µg/h sufentanil as the background dose and 3.0 µg bolus with an 18-min lockout time, and 100 mg flurbiprofen every 12 h). If required, antiemetic tropisetron (5 mg/day) or metoclopramide (10 mg/day) was intra-venously administered.

### Surgical procedure

Minimally invasive thoracoscopy and laparoscopic esophagectomy constitute the standard approach for patients with esophageal cancer of any stage [6, 8]. Briefly, the patients were placed in the left semi-prone position. Then, the thoracic esophagus was completely freed, and the lymph nodes in the para-esophageal regions were dissected. Subsequently, the stomach, abdominal esophagus, and cervical esophagus were freed, and the peri-gastric lymph nodes were dissected. A 5-cm incision was made in the center of the abdomen. Then the stomach was removed and shaped into a gastric tube, and a mechanical anastomosis was constructed from the cervical esophagus. Three-field lymph-node dissection (3-FLND) and cervical anastomosis were performed in the cases of upper esophageal cancer, and 2-FLND and intra-thoracic anastomosis were performed for mid- or lower-esophageal cancer.

### Data collection

Baseline parameters of interest, including age, sex, body mass index (BMI), American Society of Anesthesiologists status (ASA), comorbidities, information on previous abdominal or thoracic surgery, tumor location (proximal, medial, and distal; 15–23, 24–32, and 33–40 cm from the teeth, respectively), post-operative length of stay (PLOS), and information on whether it was a night surgery (operations finished or started after 8:00 P.M) were collected from the patient records. The retrieved intra-operative and post-operative factors consisted of hemodynamic characteristics, respiratory characteristics, body temperature, and information on fluid management. In-hospital mortality was defined as “all-cause” mortality associated with hospital admission for esophagectomy.

### Statistical analysis

Comparisons of the clinicopathological characteristics between two groups were performed using the Chi-squared test and Fisher’s exact test for categorical parameters, and the Student’s *t*-test or ANOVA for continuous variables. Charts were prepared using GraphPad Prism 6 (GraphPad Software, Inc., La Jolla, CA, USA). To analyze whether the intra-operative or post-operative clinical parameters influenced the risk of ETI, logistic regression models were constructed, and hazard ratios (HR) with 95% confidence intervals (CI) were estimated. Cox regression analysis was performed to analyze the factors affecting OS. Statistical analysis was performed using SPSS 23.0 (IBM Corp. Armonk, NY, USA). A two-sided *p*-value < 0.05 was considered to indicate statistical significance.

## Results

### Demographics and patient characteristics

All the 21 patients who underwent ETI after esophagectomy during the study period of four years and 21 patients who underwent only esophagectomy during the same interim were included in the study (ETI and control groups, respectively). The demographic information about the patients is summarized in Table 1. There were far more men than women in both groups, but no significant difference was found between the two groups (*p* = 0.107). The average age of the patients in the ETI group was higher than that of the control patients (*p* = 0.022). There were significantly more patients with a history of smoking in the ETI group than in the control group (*p* = 0.013). The two groups did not significantly differ in the number of patients with a history of thoracoabdominal surgery (*p* = 0.147). Other demographic characteristics, such as BMI, forced expiratory volume in the first second (FEV_1_), ASA score, tumor location, depth of invasion, and pre-operative comorbidities, were all similar between the two groups (all *p* > 0.05) (Table 1).

**Table 1:** Demographic and preoperative characteristics of patients who underwent esophagectomy for cancer

	ETI (*n* = 21)	Control (*n* = 21)	*p*-value
Male gender ^a^	17(81.0%)	21(100%)	0.107
Age (years) ^b^	64.57 ± 9.39	58.29 ± 7.62	0.022
BMI (kg/m^2^) ^b^	22.71 ± 3.76	21.76 ± 2.45	0.336
ASA^a^			
II	16(76.2%)	19(90.5%)	0.408
III	5(23.8%)	2(9.5%)	
FEV_1_ < 70%^ a^	3(14.3%)	1(4.8%)	0.599
Smoke ^a^	13(61.9%)	5(23.8%)	0.013
History of thoracoabdominal surgery ^a^	7(33.3%)	3(14.3%)	0.147
Depth of invasion^c^			0.222
T_1_	3(14.3%)	6(28.6%)	
T_2_	4(19.0%)	2(9.5%)	
T_3_	5(23.8%)	9(42.9%)	
T_4_	9(42.9%)	4(19.0%)	
Location of tumor^c^			0.802
Proximal	2(9.5%)	1(4.8%)	
middle	3(14.3%)	4(19.0%)	
distal	16(76.2%)	16(76.2%)	
comorbidities ^a^			
cardiovascular system	12(57.1%)	10(47.6%)	0.537
Respiratory system	13(61.9%)	11(52.4%)	0.533
Gastrointestinal system	10(47.6%)	6(28.6%)	0.204

Cardiovascular system: History of myocardial infarction, heart failure, cardiac arrhythmia, hypertension or (treated) coronary artery disease; Respiratory system: history of chronic bronchitis, emphysema, silicosis, pulmonary bullae, asthma; Gastrointestinal system: history of gastroenteritis, dyspepsia, reflux esophagitis, gastric ulcer, intestinal obstruction

*ETI* Emergent endotracheal intubation, *BMI* Body mass index, *ASA* American Society of Anesthesiologists, *FEV*_*1*_ forced expiratory volume in the first second

^a^Data presented as numbers or percentages, chi-square test or Fisher’s exact test

^b^Data presented as mean ± standard deviation, two-tailed Student’s t test

^c^Data presented as numbers or percentages, One way ANOVA

### Peri-operative parameters

The peri-operative characteristics of the patients are summarized in Table 2. Compared with the control group, the ETI group comprised more patients who underwent esophagectomy at night (operation finished or started after 8:00 P.M), but the difference did not reach statistical significance (33.3% vs. 57.1%; *p* = 0.121). The two groups did not significantly differ in histological characteristics (*p* = 0.659) or whether lymph-node dissection was performed (*p* = 0.533). The PACU stay time in the ETI group was significantly longer than that in the control group (*p* = 0.001).

**Table 2:** Perioperative characteristics of patients who underwent esophagectomy for cancer

	ETI (*n* = 21)	Control (*n* = 21)	*p*-value
**Night surgery**^**a**^	12(57.1%)	7(33.3%)	0.121
**Histology**^a^			
** squamous carcinoma**	17(81.0%)	19(90.5%)	0.659
** Adenocarcinoma**	4(19.0%)	2(9.5%)	
**Lymph node dissection**^a^			0.533
** Two-field**	13(61.9%)	11(52.4%)	
** Three-field**	8(38.1%)	10(47.6%)	
**hypotension**^a^	6(28.6%)	2(9.5%)	0.116
**hypertension**^a^	6(28.6%)	4(19.0%)	0.469
**Bradycardia**^a^	0(0.0%)	3(14.3%)	0.231
**Tachycardia**^a^	7(33.3%)	3(14.3%)	0.147
**Hypothermia**^a^	13(61.9%)	8(38.1%)	0.123
**Operation time (min)** ^b^	337.86 ± 51.09	319.95 ± 51.10	0.263
**PACU time (min)** ^b^	107.73 ± 58.69	58.67 ± 24.08	0.001
**Intubation off PACU**^a^	10(47.6%)	6(28.6%)	0.204
**Reintubation**^a^	3(14.3%)	0(0%)	0.231
**Fluid infusion (L)** ^b^	2.53 ± 0.65	2.34 ± 0.51	0.298
**Blood loss (ml)** ^b^	133.33 ± 57.73	123.81 ± 78.45	0.657
**Urine volume (ml)** ^b^	609.52 ± 238.55	561.90 ± 190.33	0.479

*ETI* Emergent endotracheal intubation, *PACU*  Post-anesthesia care unit

^a^ Data presented as numbers (percentages), chi-square test or Fisher’s exact test

^b^Data presented as mean ± standard deviation, two-tailed Student’s t test

Basic peri-operative vital signs, including blood pressure, heart rate, and body temperature, were not significantly different between the two groups (*p* > 0.05). Other characteristics, such as urine volume and the numbers of patients subjected to reintubation in the PACU, freed of intubation in the PACU, treated with fluid infusion, or inflicted with blood loss, were also similar between the two groups (all *p* > 0.05) (Table 2).

### Post-operative complications and clinical outcomes

The post-operative complications and clinical outcomes are summarized in Table 3. The most common post-operative complications in the ETI group were pulmonary (81.0%) and electrolyte (81.0%) disorders, followed by cardiac complications (57.1%); yet the most common post-operative complications in the control group were cardiac complications (38.1%), followed by electrolyte (33.3%) and hepatic (28.6%) disorders. The incidence of pulmonary complications in the ETI group was far higher than that in the control group (*p* < 0.001). The incidence of an anastomotic leak or electrolyte disorder in the ETI group was also higher than that in the control group (*p* = 0.014; *p* = 0.002). Additionally, the incidence of shock in the ETI group was higher than that in the control group, but the difference was not statistically significant (*p* = 0.057) (Table 3).

**Table 3:** Post-operative complications and clinical outcomes of patients who underwent esophagectomy for cancer

	ETI (*n* = 21)	Control (*n* = 21)	*p*-value
***Postoperative complications***			
Pulmonary^a^	17(81.0%)	4(19.0%)	0.000
Cardiac^a^	12(57.1%)	8(38.1%)	0.217
Anastomotic leak^a^	9(42.9%)	2(9.5%)	0.014
Hepatic^a^	9(42.9%)	6(28.6%)	0.334
Renal^a^	1(4.8%)	0(0%)	
Thromboembolic^a^	7(33.3%)	2(9.5%)	0.133
Electrolyte disorder^a^	17(81.0%)	7(33.3%)	0.002
Postoperative bleeding^a^	2(9.5%)	0(0%)	0.469
Sepsis^a^	3(14.3%)	0(0%)	0.231
Shock^a^	5(23.8%)	0(0%)	0.057
***Clinical outcomes***			
Reoperations^a^	12(57.1%)	1(4.8%)	0.000
PLOS (days) ^b^	38.17 ± 21.41	17.67 ± 9.41	0.000
Charge (*10^3^ RMB) ^b^	198.55 ± 15.94	88.75 ± 11.85	0.000
In-hospital Mortality^a^	4(19.0%)	0(0%)	
3-year OS^a^	10(47.6%)	18(85.7%)	0.018

Data are presented as number (percentage), or ratio or mean ± standard deviation

*ETI* Emergent endotracheal intubation, *PLOS* Post-operative length of stay, *OS* Overall survival

^a^Chi-square

^b^Two-tailed Student’s *t* test

The incidence of reoperation in the ETI group was far higher than that in the control group (*p* < 0.001). Additionally, the PLOS in the ETI group was far longer and the hospitalization expenses were far higher than those in the control group (both *p* < 0.001) (Table 3).

### Causes and predictors of ETI

The most common cause of intubation was refractory hypoxemia (18/21; 85.7%), followed by cardiopulmonary arrest (2/21, 9.5%), and hemorrhagic shock (1/21, 4.8%). Logistic regression analysis revealed a history of smoke (HR 6.43, 95%CI 1.39–29.76, *p* = 0.017) or long PACU stay (HR 1.04, 95%CI 1.01–1.83, *p* = 0.020) to be associated with a high risk of ETI. Night surgery (*p* = 0.125) or peri-operative hypotension (*p* = 0.203) was not found to be a risk factor for ETI (Tables 4 and 5).

**Table 4:** Causes of intubation and hospital death of inpatients who underwent emergent tracheal intubation

Characteristics	ETI (*n* = 21)
***Reasons for intubation***	
Cardiopulmonary arrest	2(9.5%)
Hemorrhagic shock	1(4.8%)
Refractory hypoxemia	18(85.7%)
***Cause of in-hospital death***	
Pulmonary embolism	2(9.5%)
Septic shock	1(4.8%)
Respiratory failure	1(4.8%)

Data are presented as numbers (percentage)

**Table 5:** Logistic analyses of intraoperative and postoperative clinical parameters potentially associated with emergent endotracheal intubation

Parameter	OR	95% CI	*p*-value
Step 1			
Age	1.07	0.97–1.17	0.161
Smoke	7.45	1.48–37.47	0.015
PACU time	1.04	1.00-1.08	0.046
Step 2			
Smoke	6.43	1.39–29.76	0.017
PACU time	1.04	1.01–1.08	0.020

*PACU* Post-anesthesia care unit

### In-hospital mortality, causes of death, and OS

**Fig. 1:**
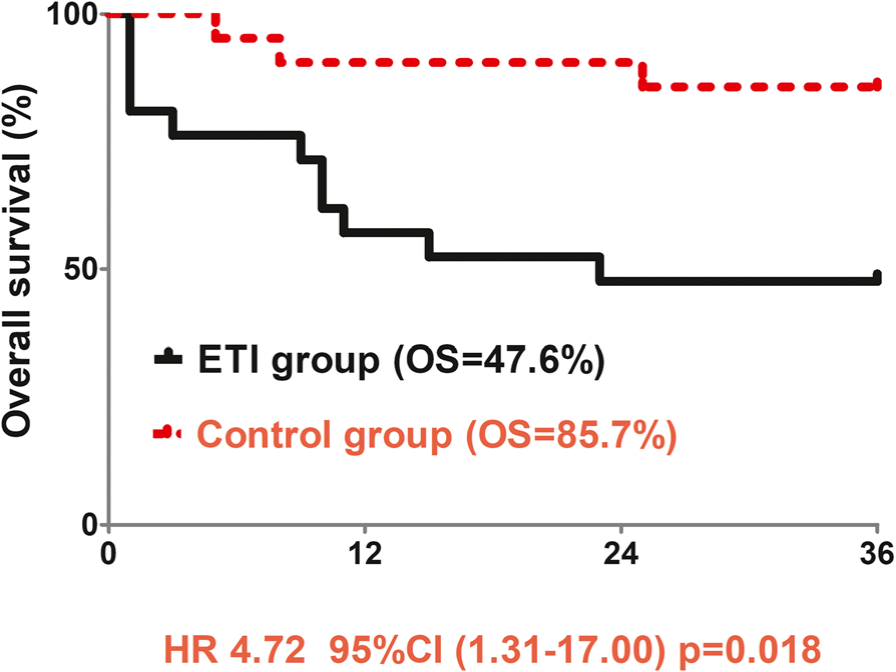
Kaplan-Meier 3-year survival curves for patients with or without ETI after esophagectomy

The in-hospital mortality in the ETI group was 19.0% (4/21). Among these cases, 2, 1, and 1 were due to pulmonary embolism, septic shock, and respiratory failure, respectively. There was no in-hospital death in the control group. The 3-year OS rate of the ETI group was estimated at 47.6% (10/21), dramatically lower than that of the control group, which was estimated at 85.7% (18/21) (HR 4.72, 95%CI 1.31–17.00, *p* = 0.018). The results of COX regression analysis indicated that ETI is an independent risk factor affecting the OS (Tables 3 and 4; Fig. 1).

## Discussion

This study found that a history of smoking and long stay time in PACU are both independently associated with high risks of ETI. Additionally, the 3-year OS of patients subjected to ETI was estimated to be far less than those without ETI, which was identified to be an independent risk factor affecting the OS of patients after esophagectomy. In this study, multiple relevant pre-operative, intra-operative, and resuscitation-related clinical parameters were extensively evaluated to assess for their association with ETI. These findings may be used to identify and reduce the post-operative complications and thereby prolong the OS in relevant cases.

This study also found that men account for the vast majority of patients with esophageal cancer, consistent with epidemiological characteristics [9]. Additionally, the patients subjected to ETI in the presented study were estimated to be older than the control patients, implying that elderly patients are more likely to undergo this serious post-operative ETI after esophagectomy than young patients. It has previously been shown that age is associated with the severity of complications after esophagectomy by an adjusted OR of 1.02 per year increase in age [10]. Naturally, elderly patients generally have reduced functional reserves of organ systems, and thus they are less tolerant to surgical trauma than young patients. Nevertheless, age by itself may not be as influential on post-operative outcomes as assumed, and treatment choice may be more important than age in old patients [10]. Thus, a pre-operative geriatric assessment is advisable to have a holistic view of the patient, which can provide an insight into the risks of post-operative complications [11, 12].

In this study, there were more patients with a history of smoking in the ETI group than in the control group. However, the two groups did not significantly differ in pre-operative FEV_1_. Patients with a history of smoking often have varying degrees of small airway damage and chronic inflammation of the trachea or bronchus, which may cause respiratory function damage under mechanical ventilation or upon surgery [13, 14]. Pulmonary function tests are not sensitive enough to detect mild-to-moderate pulmonary function impairment. Furthermore, some patients with moderate or severe pulmonary dysfunction, who were excluded from the present study, may not tolerate radical esophagectomy, and neoadjuvant therapy might be considered as an alternative treatment. In short, a history of smoking is a risk factor for post- esophagectomy ETI [15].

Inconsistent with the results of this study, a high BMI or history of thoracoabdominal surgery is generally assumed to complicate an operation and increase the incidence of post-operative complications [16]. It should be also noted that there is currently no plausible explanation for this assumption. Nevertheless, the small size of the study population may be the reason underlying this discrepancy.

A previous study has indicated that performing the surgery during off-hours, including weekends and nights, can increase the occurrence of intra-operative adverse events [17]. In the presented study, 57.1% of the patients subjected to ETI had received esophagectomy after 8:00 P.M, as opposed to 33.3% of the control patients. However, the results of small-sample regression analysis did not indicate any contribution of a night surgery to the incidence of post-operative ETI. Additionally, our previous study has indicated that ETI during off-hours is not associated with increased mortality in hospitalized patients [5].

Previous studies have identified blood loss, hypotensive events, insufficient oxygen delivery, and a need for inotropic support during esophagectomy as peri-operative risk factors for post-operative anastomotic leakage. Oxygenation of the gastric tube resulting from reduced tissue perfusion is considered one of the main causes of insufficient anastomotic healing [18]. Therefore, maintenance of blood circulation and adequate tissue oxygenation during intra-operative and post-operative periods are presumably important. Additionally, pulmonary complications can result from intra-operative hypoxemia or hypotension, which trigger the release of proinflammatory mediators and activation of leucocytes [19]. In the current study, there were no patients with severe intra-operative hypotension or hypoxemia. Regarding peri-operative mean arterial pressure measurements, intra-operative blood loss, and a need for inotropic support, no differences were observed between the ETI and control groups. In general, the influence of each specific intra-operative or post-operative parameter on the occurrence of ETI was not elucidated. Perhaps, the intensities of these factors cumulatively affect the risk of ETI. However, the incidence of post-operative pulmonary complications or anastomotic leakage was significantly higher in the ETI group than in the control group.

After the operation, every patient with an endotracheal tube was sent to the PACU. Esophagectomy requires a thoracoabdominal combined incision, which is very traumatic, and lasts for > 5 h. Some patients had a long recovery time in the PACU. The patients in the ETI group were resuscitated in the PACU in approximately 120 min, yet the control patients were resuscitated in approximately 60 min. Because of hypoxemia, hemodynamic instability, or insufficient ventilation, 47.6% of the patients subjected to ETI and 28.6% of the control patients could not be extubated in the PACU and had to be sent to the surgical ward or ICU. Three patients in the ETI group had to be reintubated after tracheal extubation in the PACU because of hypoxemia or hypercapnia. Previous studies have shown that a long duration of mechanical ventilation is associated with increased incidence of lung injury, lung infection, or regurgitation [20, 21]. Consistent with our findings, the study has found that a long stay time in PACU is another risk factor for post-esophagectomy ETI.

Strict and appropriate preoperative evaluation of patients may also be an important factor affecting the prognosis [22]. There was a specialist anesthesiologist for pre anesthesia evaluation in the hospital. For severe organ dysfunction, including cardiopulmonary function, specialist anesthesiologist could recommend: (1) Postponed the operation for further examination and treatment; (2) Changed the operation mode (patients may not tolerate radical resection of esophageal cancer to palliative resection or radiotherapy or chemotherapy); (3) For some patients with lung resection, it was the surgeon who decides the scope of surgery, other than the anesthesiologist. In the future, the pre anesthesia evaluation may be more refined, and anesthesiologists need to be more involved in the formulation of the operation plan, especially in the perioperative organ function regulation.

In this study, 57.1% of the patients subjected to ETI and 4.8% of the control patients had to undergo reoperation, including closed thoracic drainage, thoracic debridement, and esophageal reconstruction. Accordingly, the post-operative hospital stays and hospitalization costs of the patients subjected to ETI significantly increased. The in-hospital mortality of the patients subjected to ETI was 19.0%, whereas no control patient died in the hospital. The high incidence of post-operative pulmonary complications or anastomotic leakage in the ETI group may be one of the causes of the high in-hospital mortality rate post-ETI. The 3-year OS of the patients in the ETI and control groups was 47.6% and 85.7%, respectively. COX regression analysis revealed that ETI is an independent risk factor affecting the OS of patients after esophagectomy.

## Limitations

Since this was a single-center retrospective study, we could not avoid some biases resulting from incomplete patient data (e.g., some of the details of the complications were occasionally missing, or vital signs were recorded discontinuously). Another limitation of this study is the relatively low number of study patients. Thus, other possible prognostic factors may have been underestimated, and some of the prognostic factors identified in this study may have been overestimated. In addition, a longer follow-up than that of the current study is required to fully evaluate the prognosis of patients subjected to post-esophagectomy.

## Conclusion

This study indicated that a history of smoking and long stay time in PACU are both independently associated with high risks of post-esophagectomy ETI, and ETI is an independent risk factor affecting the post-esophagectomy OS.

## Data Availability

The datasets used or analysed during the current study are available from the corresponding author on reasonable request.
